# Modeling the CO_2_ separation capability of poly(4-methyl-1-pentane) membrane modified with different nanoparticles by artificial neural networks

**DOI:** 10.1038/s41598-023-36071-x

**Published:** 2023-05-31

**Authors:** Seyyed Amirreza Abdollahi, Seyyed Faramarz Ranjbar

**Affiliations:** Faculty of Mechanical Engineering, University of Tabriz, Tabriz, Iran

**Keywords:** Chemical engineering, Environmental sciences

## Abstract

Membranes are a potential technology to reduce energy consumption as well as environmental challenges considering the separation processes. A new class of this technology, namely mixed matrix membrane (MMM) can be fabricated by dispersing solid substances in a polymeric medium. In this way, the poly(4-methyl-1-pentene)-based MMMs have attracted great attention to capturing carbon dioxide (CO_2_), which is an environmental pollutant with a greenhouse effect. The CO_2_ permeability in different MMMs constituted of poly(4-methyl-1-pentene) (PMP) and nanoparticles was comprehensively analyzed from the experimental point of view. In addition, a straightforward mathematical model is necessary to compute the CO_2_ permeability before constructing the related PMP-based separation process. Hence, the current study employs multilayer perceptron artificial neural networks (MLP-ANN) to relate the CO_2_ permeability in PMP/nanoparticle MMMs to the membrane composition (additive type and dose) and pressure. Accordingly, the effect of these independent variables on CO_2_ permeability in PMP-based membranes is explored using multiple linear regression analysis. It was figured out that the CO_2_ permeability has a direct relationship with all independent variables, while the nanoparticle dose is the strongest one. The MLP-ANN structural features have efficiently demonstrated an appealing potential to achieve the highest accurate prediction for CO_2_ permeability. A two-layer MLP-ANN with the 3-8-1 topology trained by the Bayesian regulation algorithm is identified as the best model for the considered problem. This model simulates 112 experimentally measured CO_2_ permeability in PMP/ZnO, PMP/Al_2_O_3_, PMP/TiO_2_, and PMP/TiO_2_-NT with an excellent absolute average relative deviation (AARD) of lower than 5.5%, mean absolute error (MAE) of 6.87 and correlation coefficient (R) of higher than 0.99470. It was found that the mixed matrix membrane constituted of PMP and TiO_2_-NT (functionalized nanotube with titanium dioxide) is the best medium for CO_2_ separation.

## Introduction

Recently, the capture and sequestration of CO_2_ (carbon dioxide)^1,2^ as a practical tool against global warming and climate change have received significant interest. According to the literature, the CO_2_ concentration in the atmosphere since the pre-industrial era till now has dramatically increased from 280 to 420 ppm, while its maximum allowable value is 350 ppm^3,4^. On the other hand, it has been estimated that the CO_2_ concentration in the atmosphere will reach 570 ppm by the current rising level at the end of 21 century^5^. On this ground, several agreements are established to reduce CO_2_ emissions by 2050 by focusing on deploying carbon capture and storage (CCS) strategies^6^. To this end, different technologies, such as absorption^7^, adsorption^8,9^, cryogenic^10^, and membranes^11^ have been proposed. However, absorption as the most mature technology owns some serious drawbacks, including corrosion of equipment^12^, environmental side-effects^13^, and cost^14^. Cryogenic as another mature technology consumes high energy^15^. Moreover, introducing a water-stable adsorbent with high selectivity and loading capacity as well as proper heat of adsorption and reasonable cost for the large-scale application is still a serious challenge^7,16,17^. Hence, membrane technology regarding being environmentally friendly, efficient, flexible, cost, maturity, and simple is considered one of the interesting strategies for gas separation^18^ and pollution monitoring^19^. CO_2_ capture and sequestration not only is crucial for post-combustion applications related to flue gas for CO_2_/N_2_ separation but also is required for pre-combustion processes for developing renewable sources of energy, including biogas upgrading^20^ and natural gas sweetening for CO_2_/CH_4_ separation^21^. The recovered carbon dioxide is also possible to use as feedstock to synthesize value-added chemicals^22^.

Routinely, membranes are developed in natural or synthetic ways^23^, and the last one is categorized as organic and inorganic^24^. To improve the gas separation performance of conventional membranes the focus is concentrated on polymeric media^25^. To this end, different polymers, including siloxanes^26^, poly acetylenes^27^, polyimides^28^, polysulfone^29^, and basic silicon polymers^30^ are employed for different separation purposes. However, polymeric membranes still have some concerns related to their permeability^31^, selectivity^32^, and stability at high pressures^33^. Accordingly, nanocomposite membranes are fabricated by adding starch^34^, ceramic^35^, metal–organic framework^36^, carbon nanotube^37^, and nanoparticle^38–40^, to the membrane body.

On these grounds, Ahn et al. added silica nano samples as fillers to the polysulfone membrane to boost the performance of the developed mixed matrix membrane^41^. They reported inclusion of nano silica samples into the polymer structure improves the permeability. Also, Pechaf et al. applied polyimide membrane and zeolite as the MMM and assessed the permeability of He, CH_4_, CO_2_, N_2_, and O_2_^42^. They claimed the fabricated membrane increases the permeability of CO_2_ and CH_4_, while some reduction was observed for N_2_ and O_2_ permeability. Further, Ismail et al. synthesized a mixed matrix membrane using poly-ether-sulfone and Matrimid 5218 by employing Zeolite 4A^43^. The study showed adding the zeolite can improve the permeability of the membrane.

Recently, machine learning (ML) models due to their flexibility, robustness, precision, and adaptability have received significant interest in a broad range of applications from engineering to medicine^44–47^. Pattern design, model recognition, fault detection, data mining, and function estimation are some of the main applications of ML^48,49^. Recently, artificial neural network (ANN)^50^, adaptive neuro-fuzzy inference system (ANFIS)^51^, support vector machine (SVM)^52^, and genetic programming (GP)^53^ have been used in the field of membrane technology. On these grounds, Rezakazemi et al. employed the ANFIS model for molecular separation in microporous membranes^54^. In another study, Vural et al. employed ANFIS topology for estimating the performance of a proton exchange membrane fuel cell^55^. In addition, Zhao et al. employed the ANN paradigm to predict the interfacial interactions and fouling in a membrane bioreactor^56^. They declared that the radial basis function has excellent ability to predict interfacial interactions. Further, Gasós et al. trusted on the artificial neural networks to create the maps of membrane-based CO_2_ separation technology^18^. Additionally, Kazemian et al. employed the benefits of SVM and genetic algorithm (GA) methodology to develop an algorithm for the membrane helices in amino acid sequences^57^.

Despite conducting many experiments on measuring CO_2_ permeability in pure poly(4-methyl-1-pentane) (PMP) and PMP-containing mixed membranes, no correlation has already been suggested in this field. Since permeability is a crucial factor in efficient CO_2_ separation by the PMP-based membranes, a reliable model is also required for its estimation. Hence, this study applies the MLP-ANN to correlate CO_2_ permeability in pure PMP and PMP/nanoparticle mixed matrix membranes to the filler type, nanoparticle dose, and pressure. Also, the performed relevancy analysis by the MLR (i.e., multiple linear regression) clarifies the effect of these variables on the potential level of CO_2_ permeability. To the best of the authors’ knowledge, this is the first attempt to predict CO_2_ permeability in PMP-containing membranes from some easily and always available parameters. Also, the designed MLP-ANN can help engineers fabricate a PMP-based membrane and adjust the working pressure to achieve maximum CO_2_ separation in various industries including gas processing, petroleum, petrochemical, as well as biogas upgrading.

## Gathered data from the literature

As already discussed, permeability is one of the key specifications of membrane technology for gas separation, which is often experimentally measured. On the other hand, several other studies have investigated the impact of employing different nanoparticles to improve the performance of polymetric membranes to this end. Accordingly, this study has developed a robust theoretical topology to estimate the CO_2_ permeability in the pure PMP and PMP/nanoparticle mixed matrix membranes, which to the best of the authors’ knowledge is the first one in this area. In this way, the nanoparticle types, their weight percentage (wt%) in the fabricated membrane, and operating pressure are the independent variables to estimate the CO_2_ permeability in a specific membrane. Table 1 presents the main statistical features of the gathered experimental data from the literature^58–61^.

**Table 1:** Literature data for the CO_2_ separation by the PMP-nanoparticle membranes^58–61^.

Variable	Minimum	Maximum	Average	Std. deviation	Tests
Additive type	Nothing, ZnO, Al_2_O_3_, TiO_2_, and TiO_2_-NT				112
Nanoparticle dose (wt%)	0	40	10.11	9.96	112
Pressure (bar)	2	25	5.86	3.49	112
CO_2_ permeability (barrer)	18.01	570.90	199.36	120.57	112

It is noteworthy that the literature has added up to 40 wt% of four nanoparticles (i.e., TiO_2_, ZnO, Al_2_O_3_, and TiO_2_-NT) to the PMP structure to fabricate different mixed matrix membranes. Also, 112 CO_2_ permeability tests have been conducted in a pressure range of 2–25 bar. The CO_2_ permeability of 18.01-570.90 barrer was reported in the literature for the pure PMP and PMP/ZnO, PMP/Al_2_O_3_, PMP/TiO_2_, and PMP/TiO_2_-NT mixed matrix membranes^58–61^.

Since this study includes both qualitative (additive type) and quantitative (nanoparticle dose and pressure) independent variables, it is also necessary to represent the earlier quantitatively. Table 2 introduces the numerical codes used in this regard.

**Table 2:** The numerical codes used to quantitatively presentation of the filler type.

Additive name	Nothing	ZnO	TiO_2_	TiO_2_-NT	Al_2_O_3_
Additive code	0	1	2	3	4

**Figure 1:**
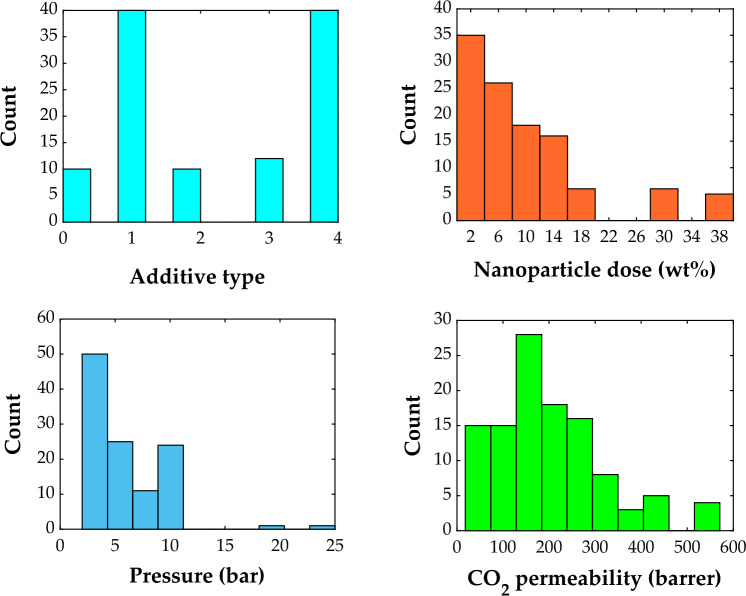
Histogram of the involved variables (additive type, nanoparticle dose, and pressure) in the modeling of CO_2_ permeability in PMP-nanoparticle membranes^58–61^.

Histograms of all independent (additive type, nanoparticle dose, and pressure) and dependent (CO_2_ permeability) variables are depicted in Fig. 1.

## Artificial neural networks

Artificial neural networks (ANNs) as a biologically inspired computational approach is a non-linear topology, which has a high capacity for data processing in the engineering area^62^. Actually, the ANNs are a reduced set of concepts derived from biological neural systems based on the simulation of data processing of the human brain and nervous systems^63^. The ANNs have already proved a robust potential for statistical analysis in the area without a broad range of experimental values regarding their flexibility and capability^62,63^. In the way of deriving an ANN paradigm, it is required to specify the main independent variables that affect the output of the process. It is worth noting that the ANNs have the potential to correlate the dependent variables with the independent ones with any degree of complexity^64^. To this end, providing a proper dataset is necessary to design a black box for the estimation of dependent factors considering defined criteria^62^. Accordingly, the obtained approach develops a signal among the input and output factors, which specifies the details in different layers related to neuron interactions.

Up to date, several ANN approaches have been developed, including multi-layer perceptron (MLP-ANN)^65^, radial basis function (RBF-ANN)^66^, cascade feedforward (CFF-ANN)^67^, general regression (GR-ANN)^68^, which the MLP-ANN is the most commonly used one. Generally, the MLP-ANN is an online learning supervised procedure that employs partial fit order together with tunable synaptic weights^69^. On these grounds, this topology was applied in this work to estimate the permeability of CH_4_ and N_2_ in PMPs. Routinely, an MLP-ANN is developed by defining three main layers, including the input layer, the hidden layer, and the output one. In this way, the input layer is derived from the raw independent (input) values after some data processing, which has already proven their high impact on the process. Then, the outcome of this layer is introduced to the hidden layer to employ statistical analysis and mathematical treatment on the data. Afterward, the outcomes of this layer are transferred to the output layer that specifies the main results of the model. It should be considered that the major mathematical processing employed on the neurons is determined by Eq. (1)^70^:

1 $$O_{j} = \sum\limits_{r = 1}^{N} {w_{jr} x_{r} + b_{j} }$$

here $b$ specifies the bias of the model, which indicates the activation thresholds for input values ($x_{r}$), and $\omega_{jr}$ is the weight coefficients of the model. Also, the net output of neurons ($O_{j}$) is received by a transfer function ($tf$) to calculate the neuron’s output^70^. In this work, the hyperbolic tangent sigmoid (Eq. 2) and logarithmic sigmoid (Eq. 3), which are among the most popular transfer functions, have been incorporated in the hidden and output layers, respectively^63,68^:

2 $$tf\left( {O_{j} } \right) = \frac{{e^{{O_{j} }} - e^{{ - O_{j} }} }}{{e^{{O_{j} }} + e^{{ - O_{j} }} }}$$

3 $$tf\left( {O_{j} } \right) = \frac{1}{{1 + e^{{ - O_{j} }} }}$$

**Figure 2:**
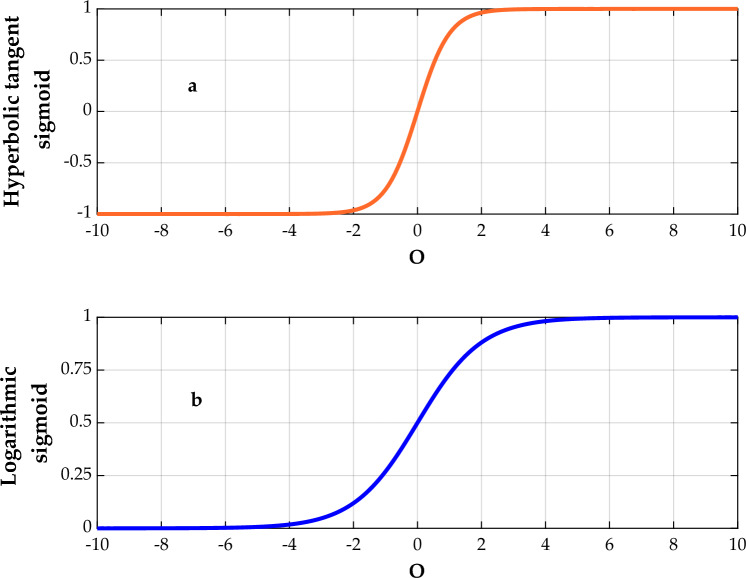
The hyperbolic tangent sigmoid (**a**) and logarithm sigmoid (**b**) transfer functions.

Figure 2a,b show the general shapes of the hyperbolic tangent sigmoid and logarithmic sigmoid transfer functions, respectively. This figure indicates that the earlier provides a value between − 1 and + 1, while the latter produces a value ranging from 0 to + 1.

To this end, it is necessary to normalize both the independent (IV) and dependent variables (DV) into the [0 1] range using Eqs. (4) and (5), respectively.

4 $$X_{i}^{j} \, = \,\left( {IV_{i}^{j} \, - \,\min \left( {IV_{i} } \right)} \right)/\left( {\max \left( {IV_{i} } \right)\, - \,\min \left( {IV_{i} } \right)} \right)\,\,\,\left\{ \begin{gathered} i = 1,\,2,\,3 \hfill \\ j = \,1,\,2,\,...,\,NoD \hfill \\ \end{gathered} \right.$$

5 $$Y^{j} \, = \,\left( {DV^{j} \, - \,\min \left( {DV} \right)} \right)/\left( {\max \left( {DV} \right)\, - \,\min \left( {DV_{i} } \right)} \right)\,\,\,\,\,\,\,j = \,1,\,2,\,...,\,NoD$$

NoD designates the number of datasets. X_1_, X_2_, and X_3_ indicate the normalized value of the additive type, nanoparticle dose, and pressure. Moreover, Y stands for the normalized CO_2_ permeability.

## Evaluation of the model’s accuracy

It is often mandatory to measure the deviation between experimental and predicted values of the dependent variable using statistical criteria. This study applies correlation coefficient (R), coefficient of determination (R^2^), summation of absolute error (SAE), mean absolute error (MAE), absolute average relative deviation (AARD), and mean squared error (MSE). Accordingly, Eqs. (6) to (11) present the formula of R, R^2^, SAE, MAE, AARD, and MSE, correspondingly^71^.

6 $$R = \sqrt {1 - \left\{ {\sum\limits_{{i = 1}}^{{NoD}} {\left( {DV^{{\exp }} - {\mkern 1mu} DV^{{cal}} } \right)_{i}^{2} } \bigg/\sum\limits_{{i = 1}}^{{NoD}} {\left( {DV^{{\exp }} - \overline{{DV^{{\exp }} }} } \right)_{i}^{2} } } \right\}}$$

7 $$R^{2} = 1 - \left\{ {\sum\limits_{{i = 1}}^{{NoD}} {\left( {DV^{{\exp }} - {\mkern 1mu} DV^{{cal}} } \right)_{i}^{2} } \bigg/\sum\limits_{{i = 1}}^{{NoD}} {\left( {DV^{{\exp }} - \overline{{DV^{{\exp }} }} } \right)_{i}^{2} } } \right\}$$

8 $$SAE = \sum\limits_{{i = 1}}^{{NoD}} {\left| {DV^{{\exp }} - DV^{{cal}} } \right|_{i} }$$

9 $$MAE = \left( {1/NoD} \right) \times \sum\limits_{{i = 1}}^{{NoD}} {\left| {DV^{{\exp }} - DV^{{cal}} } \right|_{i} }$$

10 $$AARD = \left( {100/NoD} \right) \times \sum\limits_{{i = 1}}^{{NoD}} {\left( {\left| {DV^{{\exp }} - DV^{{cal}} } \right| \big/ DV^{{\exp }} } \right)_{i} }$$

11 $$MSE = \left( {1/NoD} \right) \times \sum\limits_{{i = 1}}^{{NoD}} {\left( {DV^{{\exp }} - DV^{{cal}} } \right)_{i}^{2} }$$

The above equations need experimental ($DV^{\exp }$) and calculated ($DV^{cal}$) dependent variables as well as the average value of the $DV^{\exp }$. Equation (12) calculates this average value, i.e., $\overline{{DV^{\exp } }}$.

12 $$\overline{{DV^{{\exp }} }} = \sum\limits_{{i = 1}}^{{NoD}} {\left( {DV^{{\exp }} } \right)_{i} /NoD}$$

## Results and discussions

This section introduces the results of relevancy analysis by MLR, MLP-ANN development, and statistical and graphical investigations of the proposed model.

### Relevancy analysis by the multiple linear regression

Before constructing the MLP-ANN to estimate the CO_2_ permeability in PMP/nanoparticle membranes, the relevancy between dependent and dependent variables must be explored. The MLR is a well-known method in this field^72^. Equation (13) is a simple MLR model that correlates the normalized CO_2_ permeability ($Y^{cal}$) to the normalized values of the independent variables based on 112 experimental datasets.

13 $$Y^{cal} \, = \,0.13745\, + \,0.05607\,X_{1} \, + \,0.48741\,X_{2} \, + \,0.21084\,X_{3}$$

**Figure 3:**
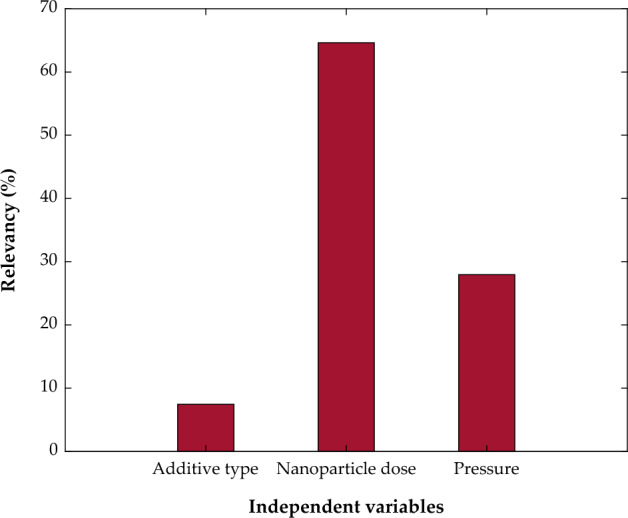
Relevancy between CO_2_ permeability in MMMs and additive type, nanoparticle dose, and pressure.

The positive sign of the X_1_, X_2_, and X_3_ coefficients suggests the direct dependency of CO_2_ permeability on the involved independent variables. Also, the coefficient magnitude shows the strength of the relationship between the dependent and independent variables. As Fig. 3 illustrates the CO_2_ permeability in PMP/nanoparticle membranes has the strongest dependency on the nanoparticle dose and the weakest dependency on the additive type.

The observed AARD = 88.24%, R^2^ = 0.40145, and SAE = 7634.84 barrer between experimental CO_2_ permeabilities and MLR predictions show that the considered problem is mainly governed by a nonlinear model.

The accuracy of indices is calculated after de-normalizing the MLR prediction for the normalized CO_2_ permeability using Eq. (14).

14 $$DV^{{cal}} = Y^{{cal}} \times \left( {DV^{{\max }} - DV^{{\min }} } \right) + DV^{{\min }} \left\{ {\begin{array}{*{20}l} {DV^{{\min }} = 18.01} \hfill \\ {DV^{{\max }} = 570.90} \hfill \\ \end{array} } \right.$$

### Nonlinear modeling by the MLP-ANN

**Figure 4:**
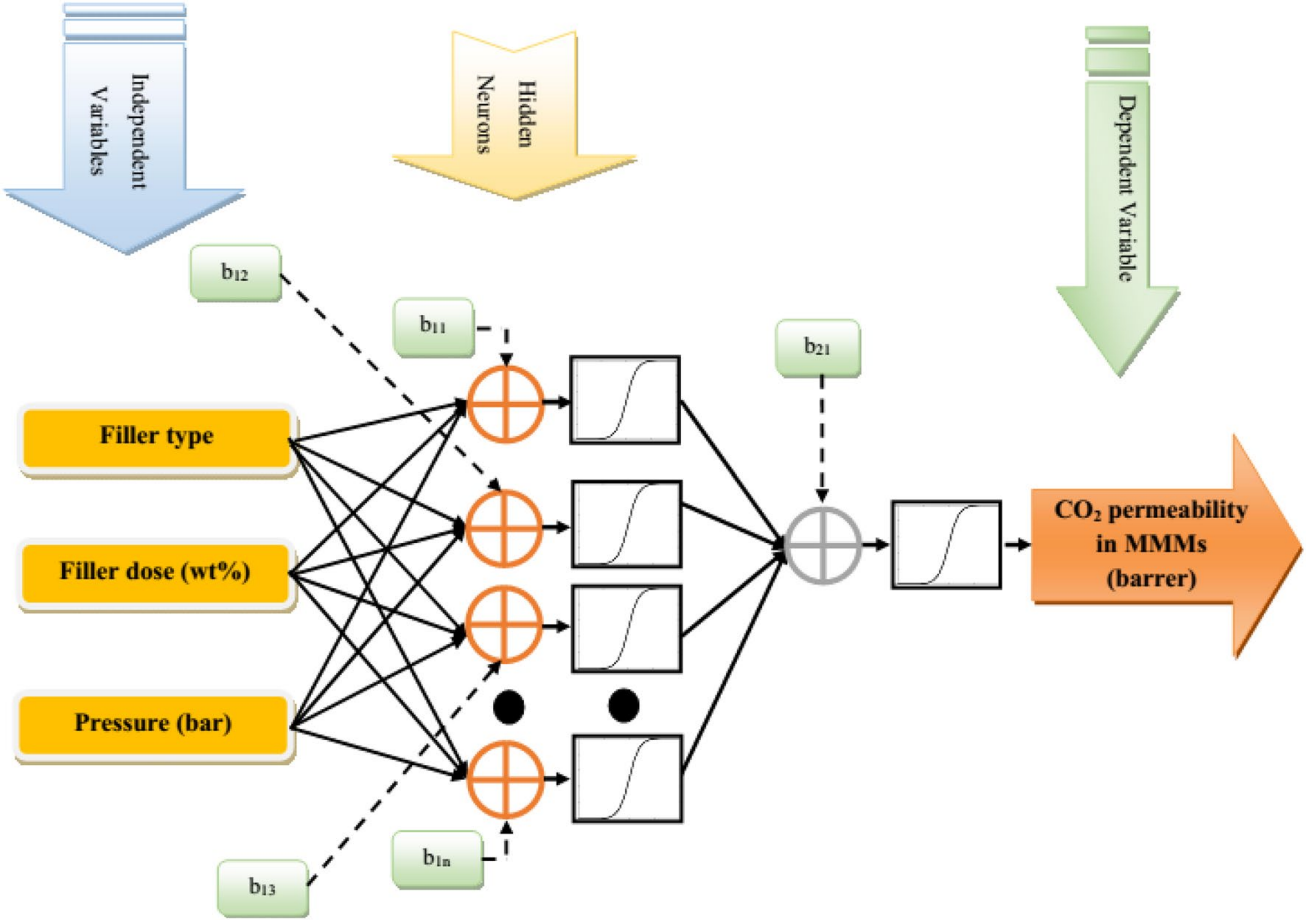
The MLP-ANN structure to simulate CO_2_ permeability in PMP/nanoparticle MMMs.

The general topology of the MLP-ANN to relate the CO_2_ permeability in PMP/nanoparticle MMMs has been shown in Fig. 4.

This stage constructs 90 MLP-ANN approaches with different numbers of hidden neurons. Indeed, these MLP-ANN models may have one to nine neurons in their hidden layers. In addition, the MLP-ANN with a specific number of hidden neurons is trained and tested 10 different times.

**Figure 5:**
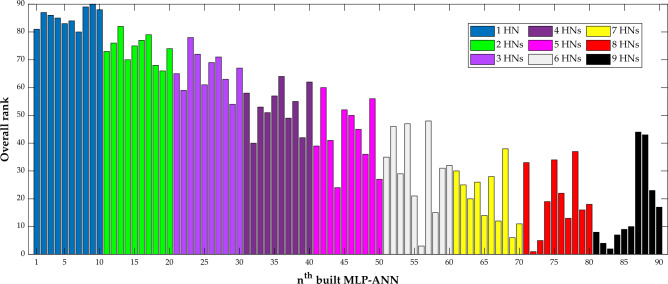
Overall ranking of the 90 constructed MLP-ANNs with 1–9 hidden neurons (10 models per each hidden neuron).

Figure 5 shows the results of ranking the 90 constructed MLP-ANN models. Generally, the MLP-ANN accuracy increases (rank decreases) by increasing the number of hidden neurons. This observation is related to the increasing MLP-ANN size as well as the number of their weights and biases. The figure indicates that the second-developed MLP-ANN with eight hidden neurons (rank = 1) is the best model for estimating the CO_2_ permeability in PMP/nanoparticle MMMs. In addition, the 9th-built MLP-ANN with only one hidden layer is the lowest accurate model (rank = 90) for the considered task.

The best MLP-ANN is applied to accomplish all subsequent analyses and the remaining 89 models are ignored.

**Figure 6:**
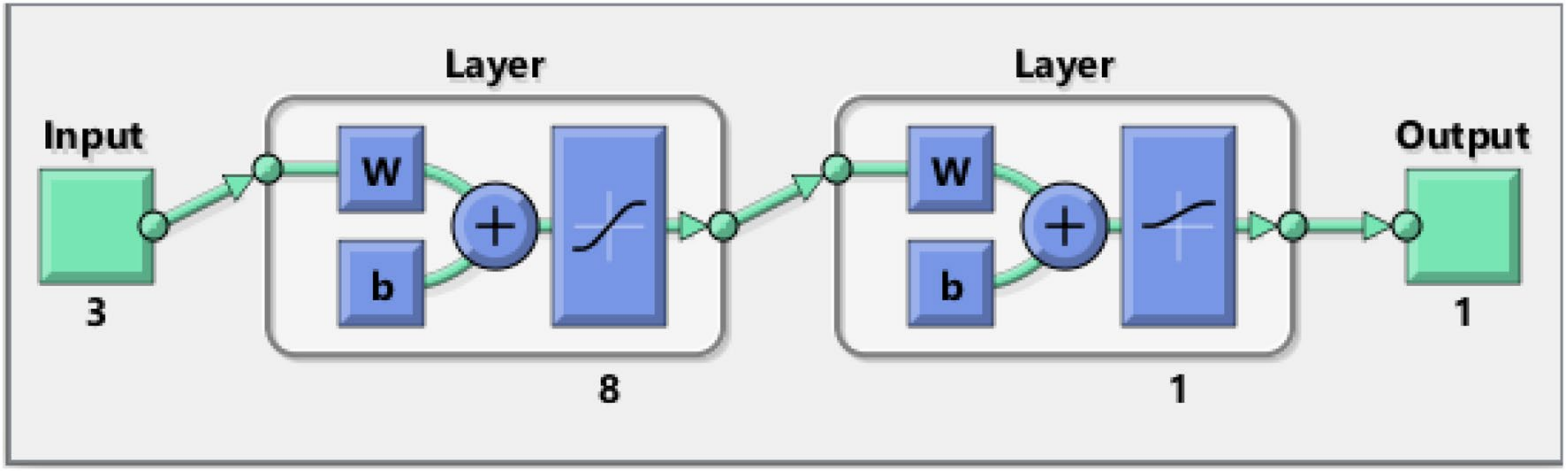
Topology of the best MLP-ANN^73^ for predicting CO_2_ permeability in PMP/nanoparticle membranes.

Figure 6 presents the general shape of the MLP-ANN approach constructed to estimate the CO_2_ permeability in MMMs. It can be seen that the MLP-ANN has only one hidden layer with eight neurons, i.e., 3-8-1 topology. The hyperbolic tangent sigmoid and logarithmic sigmoid transfer functions can also be seen in the hidden and output layers. It should be noted that the modeling phase of the CO_2_ permeability in both PMP and PMP/nanoparticle membranes is done in the MATLAB environment (Version: 2019a)^73^.

Table 3 reports the achieved accuracy of the proposed MLP-ANN in the training and testing stages. This table also shows the accuracy of the built MLP-ANN model for predicting the CO_2_ permeability of the overall datasets. Five statistical criteria (i.e., R, MAE, AARD, MSE, and SAE) have been used in this regard. All these accuracies are acceptable enough from the modeling point of view.

**Table 3:** Accuracy of the best MLP-ANN for estimating the CO_2_ permeability in MMMs.

Data group	R	MAE	AARD	MSE	SAE
Training group	0.99658	5.28	5.20%	100.54	501.84
Testing group	0.98433	15.76	6.88%	444.52	267.84
Overall data	0.99477	6.87	5.46%	152.75	769.68

### Performance checking

**Figure 7:**
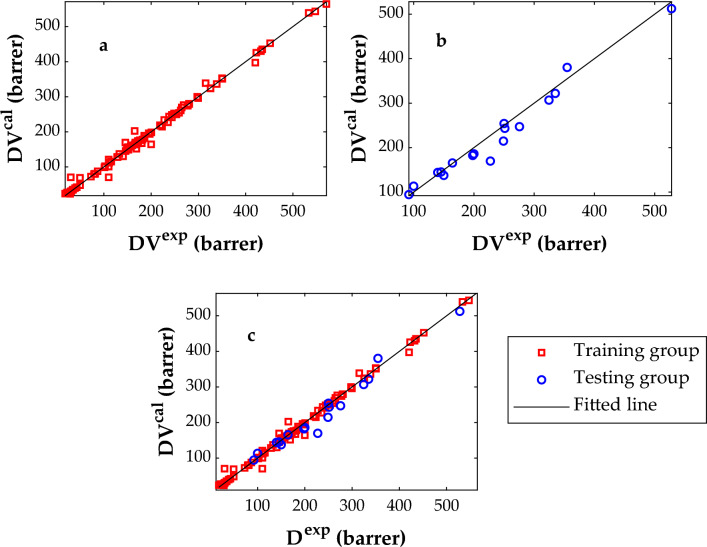
Linear correlations between experimental and calculated CO_2_ permeability in MMMs; training (**a**), testing (**b**), and overall database (**c**).

The cross-plot which graphically inspects the linear correlation between experimental and predicted values of a dependent variable is a practical method to evaluate the reliability of data-driven models. Figure 7a–c illustrate the linear correlation between experimental CO_2_ permeabilities and their associated calculated values by the MLP-ANN approach. Since both training and testing datasets are mainly located around the diagonal lines, the MLP-ANN reliability is approved by the visual inspection. Moreover, the closeness of the correlation coefficients of the training, testing, and all datasets to R ~ 1 (i.e., 0.99658, 0.98433, and 0.99477) is another indication of the MLP-ANN model.

**Figure 8:**
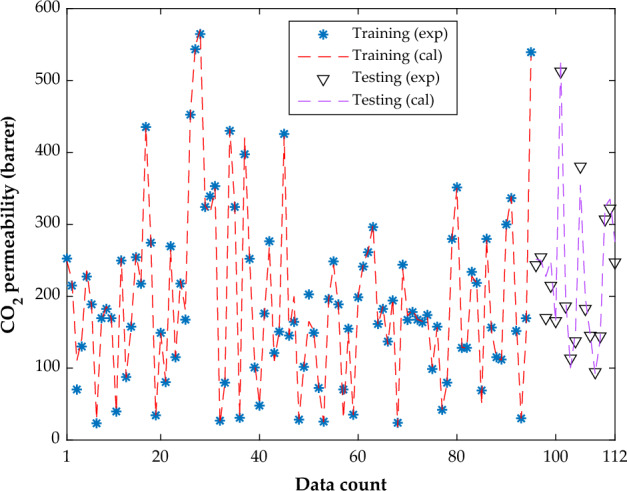
Compatibility between experimen6tal and calculated CO_2_ permeability in MMMs.

The actual and predicted CO_2_ permeabilities in the pure PMP membranes and PMP/nanoparticles MMMs in the training, as well as testing stages are depicted in Fig. 8. This analysis justifies the outstanding performance of the MLP-ANN to model both training and testing datasets. In addition, the MLP-ANN accuracy for predicting the training (MAE = 5.28, AARD = 5.20%, MSE = 100.54, and SAE = 501.84) and testing group (MAE = 15.76, AARD = 6.88%, MSE = 444.52, and SAE = 267.84) is approved by the statistical investigation. In addition, the overall values of the MAE, AARD, MSE, and SAE are 6.87, 5.46%, 152.75, and 769.68, correspondingly.

### Trend analysis

**Figure 9:**
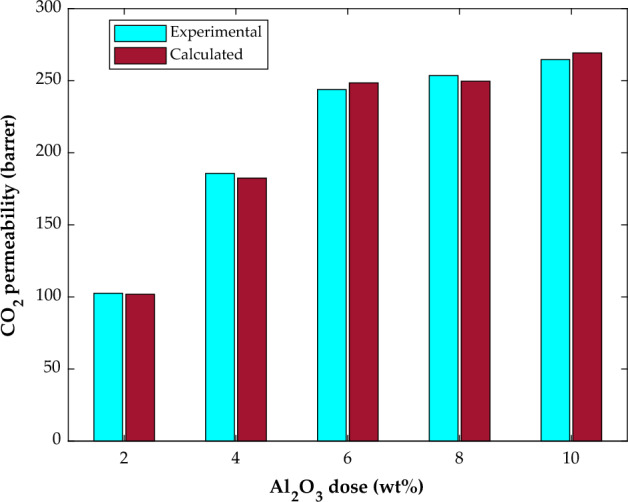
The effect of additive dose on the CO_2_ permeability in PMP/Al_2_O_3_ membranes (pressure = 10 bar).

Figure 9 explains the effect of alumina concentration on CO_2_ permeability in the PMP/Al_2_O_3_ membrane from the modeling and experimental point of view. The outstanding agreement between actual and estimated CO_2_ permeabilities in the PMP/Al_2_O_3_ MMMs can be easily found in this figure. The MLP-ANN also accurately learns the increasing effect of the filler dose on CO_2_ separation by the membrane-based process. Increasing the CO_2_ permeability in membranes by increasing the filler dose was also previously forecasted by the MLR relevancy investigation.

The literature has related this permeability improvement to the alumina-polymer interactions and pore volume increment due to the Al_2_O_3_ presence within the polymer chain^61^.

**Figure 10:**
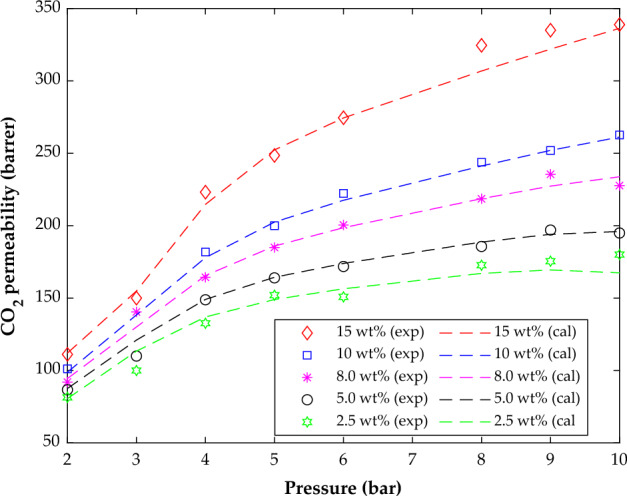
The effect of pressure on the CO_2_ permeability in PMP/ZnO membranes with different additive dosages.

The effect of working pressure on CO_2_ separation by the PMP/ZnO membranes with five nanoparticle concentration levels (2.5, 5, 8, 10, and 15 wt%) has been presented in Fig. 10. This figure displays both laboratory-measured CO_2_ permeabilities and their related MLP-ANN predictions. An excellent agreement between the experimental and modeling permeability-pressure profiles is easily observable through this investigation. The MLP-ANN also correctly identifies the pressure as well as the filler effect on CO_2_ permeability in PMP/ZnO mixed matrix membranes.

As expected, the CO_2_ permeability in the mixed matrix membranes rises by increasing the working pressure. This observation is in a direct relationship with the driving force improvement due to the pressure enhancement.

**Figure 11:**
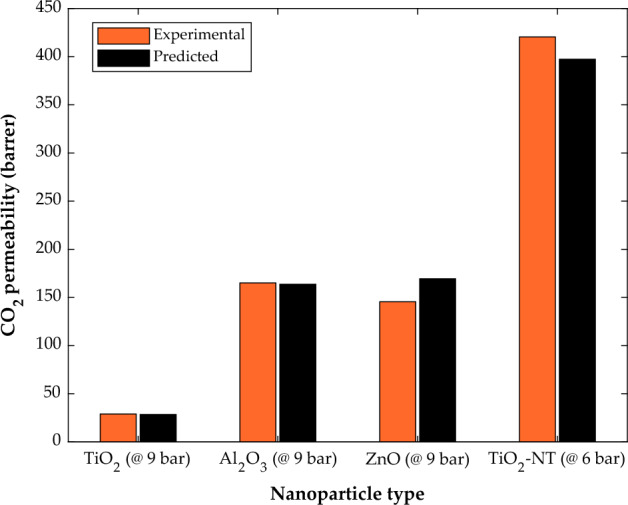
The effect of additive type on the CO_2_ permeability in PMP/nanoparticle membranes.

The effect of filler type (ZnO, Al_2_O_3_, TiO_2_, and TiO_2_-NT) on the CO_2_ separation ability of PMP-based membranes in the same working pressure is illustrated in Fig. 11. It can be seen that different fillers represent various roles in CO_2_-MMM interaction. Indeed, the PMP/TiO_2_ and PMP-TiO_2_-NT provide the CO_2_ molecule with minimum and maximum permeabilities within the membrane structure. The literature justified the higher CO_2_ permeability in PMP-TiO_2_-NT to the free volume expansion and porosity increase due to the functionalized nanoparticle presence in the membrane body^60^.

## Conclusions

This study uses a two-step methodology, i.e., multiple linear regression and multilayer perceptron artificial neural networks to simulate carbon dioxide permeability in mixed matrix membranes. The carbon dioxide permeability in pure poly(4-methyl-1-pentene) and PMP/nanoparticle membranes (i.e., PMP/ZnO, PMP/Al_2_O_3_, PMP/TiO_2_, and PMP/TiO_2_-NT) has been studied based on 112 experimental datasets collected from the literature. The multiple linear regression method applies to anticipate the dependency of the carbon dioxide permeability on the membrane composition (additive type and dose) and pressure. This method shows that the carbon dioxide permeability is directly related to all independent variables and it has the strongest correlation with the nanoparticle dose in membrane structure. The MLP-ANN is then utilized to construct a non-linear approach to estimate the carbon dioxide permeability as a function of additive type, nanoparticle dose, and pressure. This MLP-ANN with the 3-8-1 topology predicted 112 experimental carbon dioxide permeabilities in the involved MMMs with excellent accuracy (i.e., R = 0.99477, MAE = 6.87, AARD = 5.46%, MSE = 152.75, and SAE = 769.68). The modeling results clarify that the PMP/TiO_2_-NT has a better carbon dioxide separation than the PMP/ZnO, PMP/Al_2_O_3_, and PMP/TiO_2_ mixed matrix membranes. Finally, the obtained results in this work demonstrated the excellent potential of the ANN for estimating the separation factors of mixed matrix membranes for carbon capture and sequestration applications.

## Data Availability

All the literature datasets analyzed in this study are available at a reasonable request from the corresponding author (S.A. Abdollahi).
